# Spatiotemporal Variation in Groundwater Quality and Source Apportionment along the Ye River of North China Using the PMF Model

**DOI:** 10.3390/ijerph19031779

**Published:** 2022-02-04

**Authors:** Chao Niu, Qianqian Zhang, Lele Xiao, Huiwei Wang

**Affiliations:** 1 College of Geology and Environment, Xi’an University of Science and Technology, Xi’an 710054, China; niuchao@xust.edu.cn (C.N.); xiaolele@xust.edu.cn (L.X.); 2 Institute of Hydrogeology and Environmental Geology, Chinese Academy of Geological Sciences, Shijiazhuang 050061, China; whuiwei@mail.cgs.gov.cn

**Keywords:** groundwater quality, anthropogenic activities, source apportionment, water quality index, positive matrix factorization model

## Abstract

Groundwater quality deterioration has attracted widespread concern in China. In this research, the water quality index (WQI) and a positive matrix factorization (PMF) model were used to assess groundwater quality and identify pollution sources in the Ye River area of northern China. Research found that TH, SO_4_^2−^, and NO_3_^−^ were the main groundwater pollution factors in the Ye River area, since their exceeding standard rates were 78.13, 34.38, and 59.38%, respectively. The main groundwater hydrochemical type has changed from HCO_3_-Ca(Mg) to HCO_3_·SO_4_-Ca(Mg). These data indicated that the groundwater quality was affected by anthropogenic activities. Spatial variation in groundwater quality was mainly influenced by land use, whereas temporal variation was mainly controlled by rainfall. The WQI indicated that the groundwater quality was better in the flood season than in the dry season due to the diluting effect of rainfall runoff. Notably, farmland groundwater quality was relatively poor as it was affected by various pollution sources. Based on the PMF model, the main groundwater pollution sources were domestic sewage (52.4%), industrial wastewater (24.1%), and enhanced water–rock interaction induced by intensely exploited groundwater (23.6%) in the dry season, while in the flood season they were domestic sewage and water–rock interaction (49.6%), agriculture nonpoint pollution (26.1%), and industrial wastewater and urban nonpoint pollution (23.9%). In addition, the mean contribution of domestic sewage and industrial sewage to sampling sites in the dry season (1489 and 322.5 mg/L, respectively) were higher than that in the flood season (1158 and 273.6 mg/L, respectively). To sum up, the point sources (domestic sewage and industrial wastewater) remain the most important groundwater pollution sources in this region. Therefore, the local government should enhance the sewage treatment infrastructure and exert management of fertilization strategies to increase the fertilizer utilization rate and prevent further groundwater quality deterioration.

## 1. Introduction

Groundwater is a vital source of water for drinking, agriculture and industry, especially in arid and semi-arid areas [1]. However, with population growth and rapid industrialization, the quality of groundwater has deteriorated in recent years [2,3,4]. Groundwater quality has serious impacts on human and ecological health. Several studies have reported that high nitrate concentrations in drinking water were associated with the risk of “blue baby syndrome” [5,6]. In addition, drinking water containing high sulfate level can enhance mercury methylation [7].

The groundwater quality is largely affected by both the natural processes (such as hydrogeological conditions, lithology, groundwater–rock interaction, and the quality of water recharge) and anthropogenic activities (such as domestic sewage, industrial wastewater, agricultural fertilizers and pesticides, and over-exploitation of groundwater) [8,9,10]. Studies have indicated that anthropogenic pollutants such as chemical fertilizer, domestic sewage, seepage from landfills, and manure are the main sources of groundwater contamination [2,11,12]. Excessive stormwater runoff and irrigation water carried phosphorus, ammonia, and chloride infiltrates into groundwater, resulting in groundwater quality degradation [13,14]. Rapid urbanization and industrialization are additional major reasons for groundwater quality degradation [4,6].

Identifying the main pollution sources and formulating targeted preventive and control measures are effective tools to prevent the deterioration of groundwater quality. Several receptor models are currently used for apportioning sources in the environment. Among these, the absolute principal component score/multiple linear regression (APCS/MLR) model, the positive matrix factorization (PMF) model and the Unmix model have proven to be useful tools in source apportionment studies [15,16,17,18]. The PMF model has one important advantage; that is, that it weighs the uncertainty of each data point and applies a nonnegative constraint to the data, thereby ensuring that the source contributions are always positive [19,20]. Consequently, it has been recommended by the U.S. Environmental Protection Agency (USEPA) as a general apportionment modeling tool. This PMF model is widely used in atmospheric [21,22] and soil [15,23] studies to apportion pollution sources. Nowadays, it has been used to identify pollution sources in the water environment [19,24].

The Ye River is a tributary of the Hutuo River located in the southwest of the Hebei Province, China. It is a mountain river originating in the Miao River in the Shouyang County of the Shanxi Province. This river flows from the southwest to northeast and empties into the Huangbizhuang Reservoir at Pingshan County. Groundwater is the main source for drinking water, agriculture, and industry in this area. However, with rapid urbanization and industrialization, the groundwater quality has been increasingly affected by human activities [13]. Nevertheless, relatively few studies have investigated groundwater quality and apportioned pollution sources in this region. 

This study had the following three objectives: (1) To determine the spatial and seasonal variations in groundwater quality along the Ye River; (2) to assess the groundwater quality using the water quality index (WQI); (3) to identify the major groundwater pollution sources and quantify the proportional contributions using the PMF model. Collectively, these results will aid in the development of effective water-quality protection strategies and utilization of groundwater resources for this and other mountain river areas with different anthropogenic influences. 

## 2. Materials and Methods

### 2.1. Description of the Study Area

The Ye River is located in the North China Plain. The study area spans from Yangquan County (Shanxi Province) to the Huangbizhuang Reservoir in Pingshan County (Hebei Province). The Ye River basin inclines from southwest to northeast and covers approximately 600 km^2^, and the total population is about 1.2 million. The study region has a semi-humid and semi-arid monsoon climate, with an average annual precipitation of 500 mm (mostly falling from May to September) and a mean annual temperature of 20 °C [24]. Rainfall was 390.9 mm in 2018, and the rainfall in the rainy season was 344.4 mm. The average flow of Ye River is 7.79 m^3^/s and 1.95 m^3^/s in the rainy season and dry season, respectively [25]. The main hydrochemical type of the Ye River is SO_4_·HCO_3_-Ca(Mg) [26].

**Figure 1:**
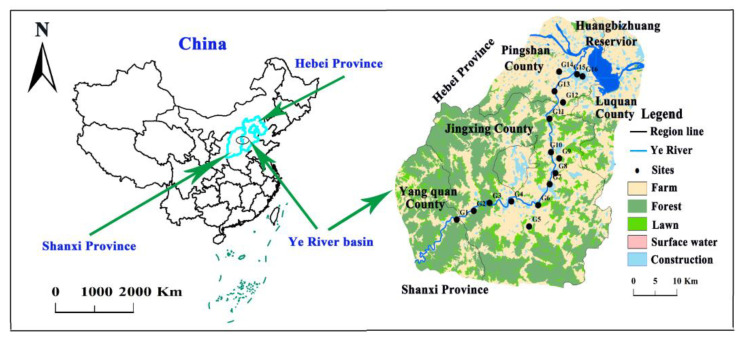
Groundwater sampling sites in the Ye River area.

The main land use types include farmland (39.1%), forest land (41.3%), grassland (13.2%), and construction land (5.2%), along with surface water bodies (1.2%) (Figure 1). In this region, the primary crops are wheat and corn. Nitrogen fertilizer is the primary agricultural fertilizer (mainly including urea, compound fertilizer, and manure). The main method of agricultural irrigation is flood irrigation. The main industrial types in the study area are coal mines, coal washing plants, metallurgy, machinery-manufacturing plants, cement plants, and power plants.

The main aquifer forms part of the Quaternary aquifer system of the Hebei Plain and has an elevation that ranges between 53 and 195 m above the sea level [13]. The main minerals of the aquifer in this region are the limestone and dolomitic limestone. Its lithology consists of gravel, pebbles, coarse sand, and fine sand [11]. The principal type of groundwater in this basin is porous aquifer, and fractured aquifer is only distributed in mountainous areas. Following the topography, the groundwater flows from southwest to northeast. In this region, the aquifer has relatively high hydraulic conductivity (k = 27.5–70.2 m/d) and the horizontal flow rate is estimated at 4 m/day [13]. Thus, groundwater is susceptible to pollutants. The groundwater is mainly recharged by precipitation, river inputs, and irrigation return, while manual exploitation is the main discharge mode. The depth of the groundwater table ranges from 3.2 to 23.4 m (the mean depth is 13.1 m). 

### 2.2. Sample Collection and Analysis

Groundwater samples were collected along the Ye River in April 2018 (dry season) and August 2018 (flood season), and comprised 16 sampling sites (According to the distance from the river (within 1 km), the depth of the groundwater table (<50 m) and consideration of different land use types, 16 groundwater sampling wells were chosen, including 8 wells in village area, 3 wells in county area, and 5 wells in farmland area) (Figure 1). All the samples were collected in porous aquifer. All the wells chosen for groundwater sampling are commonly used for domestic and/or agricultural purposes, and the mean depth of wells is 26.9 m (ranging between 12 and 50 m). Before collecting samples, these wells were purged for 5–10 min until the pH and EC of the groundwater were stable. Groundwater samples were extracted by pumping water from the wells. The pH and dissolved oxygen (DO) values were measured in the field using a multiparameter instrument (HACH HQ40d, USA). All the water samples were filtered through 0.45 μm membrane filters and then stored in 500 mL and a 1.5 L high-density polyethylene sampling bottles for water quality parameter analysis. Samples without preprocessing were used for anion analysis, while those used for cation and metal analysis were acidified with HCl to pH < 2.

The determination of anions (nitrate (NO_3_^−^), nitrite (NO_2_^−^), sulfate (SO_4_^2−^), and chloride (Cl^−^)) was carried out using a spectrophotometer (Perkin-Elmer Lambda 35, Waltham, MA, USA). The cations (potassium (K^+^), sodium (Na^+^), calcium (Ca^2+^), magnesium (Mg^2+^) and ammonia (NH_4_^+^)) and metals (iron (Fe) and manganese (Mn)) were measured using an inductively coupled plasma-emission spectrometer (Agilent 7500ce ICP-MS, Tokyo, Japan); total dissolved solids (TDS) were measured using gravimetric methods, and the chemical oxygen demand (COD) was measured using alkaline permanganate oxidation. Total hardness (TH) was measured by the ethylene–diamine–tetraacetic acid (EDTA) titration method. The water chemistry was analyzed at the laboratory of the Groundwater Mineral Water and Environmental Monitoring Center at the Institute of Hydrogeology and Environmental Geology of the Chinese Academy of Geological Sciences. The chemical analysis results of all groundwater samples were examined by anion–cation balance test to ensure the relative error was less than ±5%. 

### 2.3. Data Analysis 

#### 2.3.1. Positive Matrix Factorization (PMF) Model

In this study, EPA PMF (version 5.0) was used to apportion the dominant pollution sources of groundwater in the Ye River area. The model can be expressed as follows:

(1) $$x_{ij}=\overset{p}{\underset{k=1}{\sum }}g_{ik}f_{kj}+e_{ij}$$

where *x_ij_* is the concentration of the *j*th water quality parameter in the *i*th sample; *g_ik_* is the contribution of the *k*th source for *i* number of samples; *f_kj_* is the concentration of the *j*th water quality parameter in the *k*th source. The residual error matrix *e_ij_* is obtained by minimizing the object function *Q*:

(2) $$\;Q=\overset{n}{\underset{i=1}{\sum }}\;\overset{m}{\underset{j=1}{\sum }}{\left(\frac{e_{ij}}{\mu_{ij}}\right)}^{2}$$

In this equation, *μ_ij_* is the uncertainty in the *x_ij_* measurement, which is calculated from the method detection limit (MDL) and the standard deviations (SDs) of the surrogate standards. When the concentration of a water quality parameter was ≤MDL, the uncertainty was calculated as:

(3) $$Unc=\frac{5}{6}\times MDL$$

Otherwise, it was calculated as:

(4) $$Unc=\sqrt{{\left(\sigma +c\right)}^{2}+MDL^{2}}$$

where *σ* is the relative SD and *c* is the level of the water-quality parameter. The EPA PMF 5.0 model was used in this study.

In this study, concentration data (including 14 water quality parameters for 16 water samples) and uncertainty data files (including sampling and analytical errors) were used as the input data for the PMF model to apportion the source contributions to groundwater quality in the Ye River area. Because the PMF model exhibits rotational ambiguity, the number of factors and the Fpeak values must be tested many times for different initial seeds to determine the variability in the PMF analysis. Different values of the rotational parameter Fpeak (between −1.5 and +1.5, in steps of 0.1) were explored. When the number of factors was set at 3 and the Fpeak was −0.1 for dry and flood seasons, the runs of the PMF model were the best (the robust *Q* value was lowest (24.69 and 15.78 for dry and flood seasons, respectively)) and passed the bootstrap test. 

#### 2.3.2. The Water Quality Index (WQI)

In this study, the WQI was used to assess the groundwater quality of the Ye River area. The WQI was calculated by assigning a weight (*W_i_*) to each water-quality indicator according to its relative importance in the overall quality of surface water for drinking purposes. Water-quality standards mainly referred to the Grade III standard for groundwater quality in China [27]. If this standard lacked a given indicator, we referred to the World Health Organization (2011) [28] standards. The assigned weight (*W_i_*) and the relative weight (*RW_i_*) for each indicator are given in Table 1. The calculated WQI values were classified into five categories: excellent water (WQI < 50), good water (WQI = 50–100), poor water (WQI = 100.1–200), very poor water (WQI = 200.1–300), and unsuitable for human consumption (WQI > 300) [29].

The WQI was calculated as follows:

(5) $$RW_{i}=\frac{W_{i}}{\sum_{i=1}^{n}W_{i}\;}$$

(6) $$\;Q_{i}=\frac{C_{i}}{S_{i}}\times 100$$

(7) $$\;SI_{i}=W_{i}\times Q_{i}$$

(8) $$\;WQI=\sum SI_{i}$$

where *Q_i_* is the quality rating, *C_i_* and *S_i_* represent the concentration (mg/L) and water quality standard of each water quality parameter, respectively, and *SI_i_* is the subindex of the *i*-th parameter.

## 3. Results and Discussion

### 3.1. Groundwater Quality Properties of the Ye River Area

Groundwater quality data are given in Table 2. The groundwater pH was neutral to mildly alkaline (ranging from 6.91 to 7.87, mean: 7.37) and all samples met the Grade III standard for groundwater quality in China [27]. The dissolved oxygen (DO) varied in the range of 2.67–9.45 mg/L, with a mean value of 6.62 mg/L. The mean groundwater TDS value was 866.80 mg/L and 31.25% of the samples surpassed the Grade III standard for groundwater quality in China [27]. The NH_4_^+^ concentrations in groundwater in two seasons were below the detection limit (BDL: detection limit = 0.04 mg/L). The mean cation concentrations were as follows, in decreasing order: Ca^2+^ (186.08 mg/L) > Na^+^ (42.19 mg/L) > Mg^2+^ (39.27 mg/L) > K^+^ (2.22 mg/L) > Fe (0.129 mg/L) > Mn (0.006 mg/L) The mean anion concentrations were: HCO_3_^−^ (312.32 mg/L) > SO_4_^2−^ (216.47 mg/L) > NO_3_^−^ (134.60 mg/L) > Cl^−^ (96.38 mg/L). The SO_4_^2−^, NO_3_^−^, Cl^−^ and Fe accounted for 34.38, 59.38, 9.38, and 6.25% of samples that surpassed the Grade III standard for groundwater quality in CHina [27]. Notably, the mean TH concentration reached 626.99 mg/L and 78.13% of the samples surpassed the Grade III standard for groundwater quality in China [27]. The above results show that the mean concentrations and exceeding standard rates of TH, SO_4_^2−^, and NO_3_^−^ were very high along the Ye River, indicating that its groundwater quality was generally affected by anthropogenic activities [30]. This result is consistent with previous studies. For example, researchers found that the main pollution factors of groundwater were TH, SO_4_^2−^, and NO_3_^−^ in the Hutuo River alluvial–pluvial fan [18]. Scholars also found that the groundwater in Songyuan City, Northeast China, has been affected by anthropogenic activities, resulting in mean TH and nitrate concentrations exceeding drinking water quality standards [31]. 

### 3.2. Groundwater Quality Assessment by Using Water Quality Index (WQI)

The WQI classification of groundwater quality for the different seasons along the Ye River is shown in Table 3. The WQI ranged from 48.4 to 138.4. In the dry season, 6.2% of the groundwater samples were graded as excellent, 56.3% as good, and 37.3% as poor. In the flood season, 6.2% of the groundwater samples were graded as excellent, 87.5% as good, and only 6.3% as poor. Overall, the groundwater quality was better in the flood season than in the dry season, possibly due to the diluting effect of rainfall runoff on pollutants [18]. In addition, the sites with the worst water quality were farmland (accounting for 66.7 and 100% of the sites with poor water quality in the dry and flood seasons, respectively). This may be due to the fact that the farmland was mainly located near villages, and its groundwater quality may have been affected by the presence of mixed pollution sources such as domestic sewage, fertilizer, and manure [12].

### 3.3. The Hydrochemical Characteristics of the Groundwater in the Ye River Area

**Figure 2:**
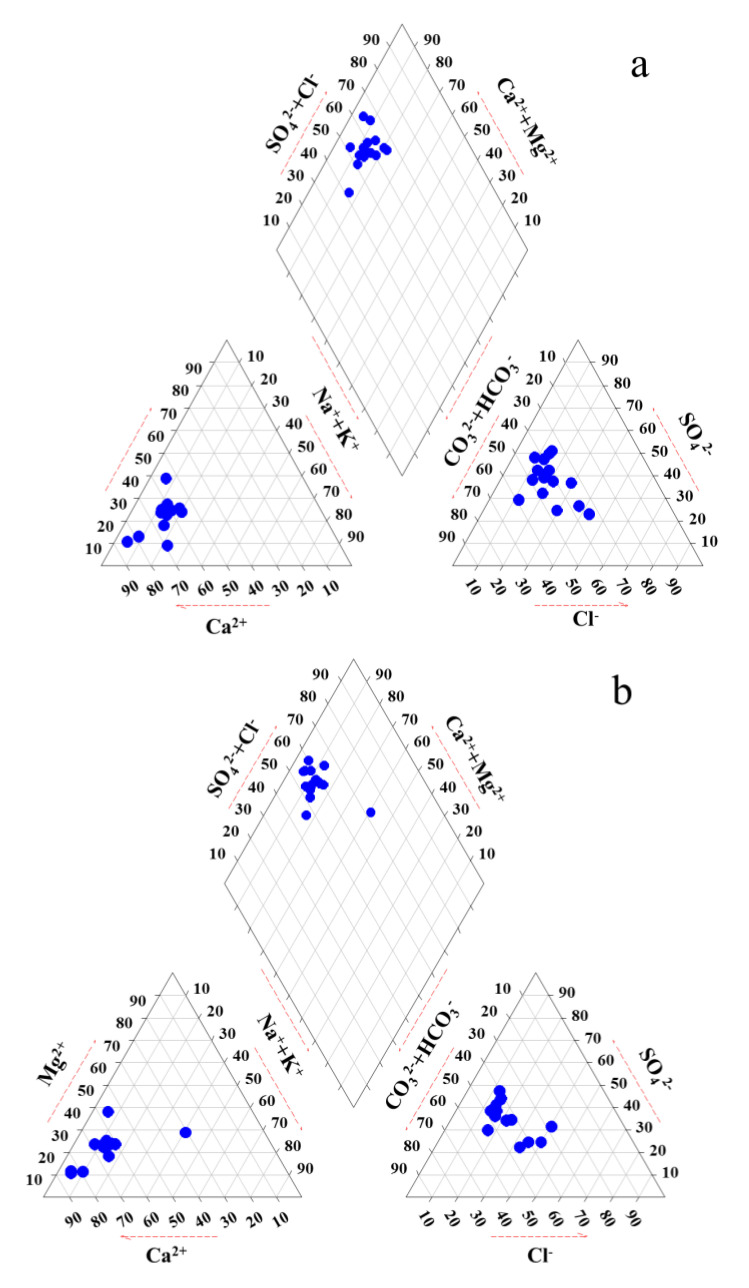
Piper diagram showing the chemical composition of the groundwater in the dry (**a**) and flood (**b**) season.

The hydrochemical components of groundwater are closely associated with the type and characteristics of strata lithology, as well as with the physical and chemical interactions occurring in the groundwater system [32,33]. In this study, the main minerals of the aquifer in this region are the limestone and dolomitic limestone. Thus, rainwater displaces a large amount of HCO_3_^−^, Mg^2+^ and Ca^2+^ from the strata in the process of replenishing groundwater. Therefore, the main hydrochemical type of groundwater in this area is mainly HCO_3_-Ca(Mg). A previous study found that the groundwater chemical type in the Shijiazhuang region was HCO_3_-Ca(Mg) before the 1950s [4]. In this study, the main groundwater chemical type was HCO_3_·SO_4_-Ca(Mg) in the Ye River area. As shown in Figure 2, the HCO_3_·SO_4_-Ca(Mg) chemical type accounted for 87.5 and 75.0% of groundwater samples in the dry and flood seasons, respectively, while the proportions of Cl-type groundwater were 18.6 and 31.3%, respectively. Similar results were reported in a previous study by Ren et al. (2020). They reported that the main hydrochemical types of groundwater were HCO_3_·SO_4_-Ca and HCO_3_·SO_4_-Ca·Mg, and Cl-type water also accounted for certain proportions in this study area [34]. It is worth noting that the hydrochemical type of site 13 is Cl-Na type in the flood season (Figure 2b), indicating that the site was severely affected by domestic sewage from the village. These data indicated that the groundwater quality in the Ye River area had undergone marked deterioration due to intensive human activities.

### 3.4. The Spatiotemporal Pattern of Groundwater Quality in the Ye River Area

**Figure 3:**
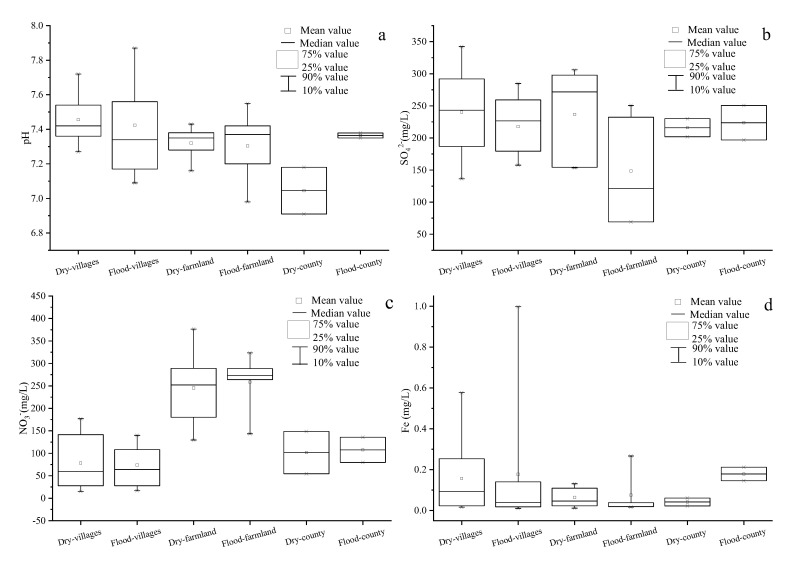
Spatial–temporal variations of (**a**) pH; (**b**) SO_4_^2−^; (**c**) NO_3_^−^, and (**d**) Fe in groundwater of the Ye River area (The number of samples in Figure 3a–d are all 32).

In this study, pH, NO_3_^−^, SO_4_^2−^ and Fe were selected to assess the spatial and temporal variation in the groundwater quality. As shown in Figure 3a,b,d, no obvious spatial variation was observed in the mean pH, SO_4_^2−^, and Fe values. However, the mean groundwater NO_3_^−^ concentration was higher in the farmland area than in the villages and county area (Figure 3c), possibly because the farmland area may have been affected not only by domestic sewage but also by agricultural fertilizers. Studies have shown that land use has an important effect on groundwater nitrate pollution [6]. In addition, land use changes may also affect the quality of the Ye River water. A previous study demonstrated that land use changes may lead strong impacts on the quality of river water [35]. This problem needs to be addressed in future studies.

In the Ye River area, temporal variations in the groundwater quality are likely to be mainly influenced by rainfall. As shown in Figure 3, no obvious temporal variation in pH was detected in the villages and farmland region (Figure 3a), which is likely because pH can be affected by multiple factors [36]. However, in the county region, pH in the flood season was slightly higher than in the flood season. The mean SO_4_^2−^ and Fe concentrations were higher in the dry season than in the flood season (except for Fe in the county region) (Figure 3b,d), which may be closely related to the dilution effect of rainfall [37]. However, the mean NO_3_^−^ concentration in the farmland area in the flood season was marginally higher than that in the dry season (Figure 3c). This may be due to the fact that rainfall runoff carries large amounts of agricultural fertilizer, which infiltrates the groundwater.

### 3.5. Identifying the Groundwater Pollution Sources Using the PMF Model

Three factors were identified in the dry and flood seasons using the PMF model. As shown in Table 4, in the dry season, Factor 1 explained 52.37% of the total water quality parameters and was associated with relatively high concentrations of TDS, K^+^, Na^+^, Ca^2+^, Mg^2+^, SO_4_^2−^, NO_3_^−^, Cl^−^, COD, and TH. Nitrate in groundwater may be mainly derived from chemical fertilizer, domestic sewage, industrial wastewater, soil nitrogen, and atmospheric deposition [38]. Domestic sewage may be the main source of NO_3_^−^ pollution in groundwater in the dry season. In the Ye River basin, especially in mountain areas, most villages do not have a constructed network of sewage pipes, and their domestic sewage drains directly into the nearby river. Previous study has confirmed that there is a close hydraulic connection between groundwater and river water in the region, and the relationship between them is river water to replenish groundwater [26]. Furthermore, the formation lithology in this area is coarse. Thus, domestic sewage seeped into the aquifer easily. In the dry season, there is relatively little rainfall (the rainfall was 46.5 mm in 2018), and the NO_3_^−^ concentration (8.94 mg/L) in rainfall is lower [11]; consequently, atmospheric deposition was not a main source of groundwater NO_3_^−^. Furthermore, in the dry season, chemical fertilizers may not have a significant effect on groundwater nitrate levels because they could not permeate into the groundwater in the absence of rainfall runoff and agricultural irrigation (Agricultural irrigation is seldom carried out in this time (January–April) in the study area). Chloride in groundwater can originate both from human activities (such as domestic sewage, industrial wastewater, chemical fertilizers, and road deicing salt) and natural sources (such as oceans, atmospheric deposition, and the weathering of evaporite rocks (halite)) [39]. In the Ye River basin, the higher concentration of Cl^−^ in groundwater may have originated primarily from domestic sewage as chloride fertilizer was rarely applied, and the Cl^−^ concentration in rainfall (2.28 mg/L) was low [11]. Furthermore, this region is far from the sea. Road deicing salt is mainly used for urban roads and it does not directly affect the groundwater in the Ye River area. This indicates that Cl^−^ also mainly originated from domestic sewage. Based on the above analysis, Factor 1 represents domestic sewage pollution (point source).

In the dry season, Factor 2 explained 24.12% of the total water-quality parameters, and it was associated with relatively high concentrations of Fe and Mn and moderate concentrations of SO_4_^2−^. Higher Fe and Mn concentrations are indicative of pollution by metals and metallic compounds and they could come from industrial effluents [40]. Indeed, the G10 site is located near an industrial park and has the greatest concentration of Fe (0.577 mg/L) and Mn (0.045 mg/L). High SO_4_^2−^ concentrations in groundwater are thought to originate from both natural and anthropogenic sources, such as atmospheric deposition, the weathering of sulfide-bearing minerals and evaporite minerals, fertilizer, and domestic and industrial wastewater [41,42]. In the Ye River basin, domestic sewage and industrial wastewater were likely the greatest sources of SO_4_^2−^. This is because the domestic sewage was discharged untreated, and there were several coal mines and coal washing plants located near the Ye River. The wastewater from coal washing was directly discharged into the Ye River, and the wastewater would inevitably have infiltrated into the groundwater. Chemical fertilizers and rainfall were not the main sources of SO_4_^2−^ in groundwater, because sulfur fertilizer was rarely applied and the SO_4_^2−^ concentration in rainfall was low (5.89–37.9 mg/L) [43]. Considering that Factor 1 stands for domestic sewage pollution, Factor 2 is accordingly considered to denote industrial wastewater pollution (point source).

In the dry season, Factor 3 explained 23.51% of the total water-quality parameters. This factor is associated with relatively high pH levels and high concentrations of HCO_3_^−^, and moderate concentrations of TDS, TH, and Ca^2+^. Studies have reported that higher concentrations of HCO_3_^−^, TH, and Ca^2+^ in groundwater may result from enhanced water–rock interactions and accelerated rock dissolution (e.g., limestone and dolomite) [44]. In the Ye River basin, groundwater has been intensely exploited due to the massive use of water in industry and agriculture, which enhanced cation exchange processes, leading to the increase in TH and Ca^2+^ levels in the groundwater. In the Ye River basin, the higher concentration of HCO_3_^−^ and Ca^2+^ was mainly due to the dissolution of limestone, expressed as Equation (9)

CaCO_3_ + H^+^ = Ca^2+^ + HCO_3_^−^ (9)

Therefore, Factor 3 represents enhanced water–rock interaction induced by intensely exploited groundwater.

In the flood season, Factor 1 explained 52.37% of the total water-quality parameters and was associated with relatively high pH levels and concentrations of Na^+^, Ca^2+^, Mg^2+^, SO_4_^2−^, NO_3_^−^, Cl^−^, HCO_3_^−^, and TH, as well as moderate concentrations of TDS and K^+^. Thus, Factor 1 was consistent with domestic sewage and water–rock interactions. Factor 2 explained 26.12% of the total water-quality parameters and was associated with relative greater concentrations of TDS and K^+^, and moderate concentrations of NO_3_^−^. As mentioned above, NO_3_^−^ in groundwater can originate from chemical fertilizer [33]. In the flood season, rainfall runoff lixiviates chemical fertilizer into groundwater, thereby increasing its NO_3_^−^ concentration. In addition, agricultural runoff has been reported to contain large amounts of ions (such as K^+^) [45]. Therefore, Factor 2 represents agricultural nonpoint pollution. Factor 3 explained 23.94% of the total water-quality parameters and was associated with relatively greater concentrations of Fe, Mn, and COD, and moderate concentrations of SO_4_^2−^. As discussed in the previous paragraph, the Fe, Mn, and SO_4_^2−^ in the groundwater were mainly derived from industrial wastewater. However, COD in groundwater may also originate from road runoff (urban nonpoint pollution), and COD has been reported to be a major pollutant in urban roads [46]. Accordingly, Factor 3 is considered to denote to industrial wastewater and urban nonpoint pollution.

### 3.6. Source Contribution Using the PMF Model

#### 3.6.1. Estimated Contribution (mg/L) of Each Source to 16 Sampling Sites

**Figure 4:**
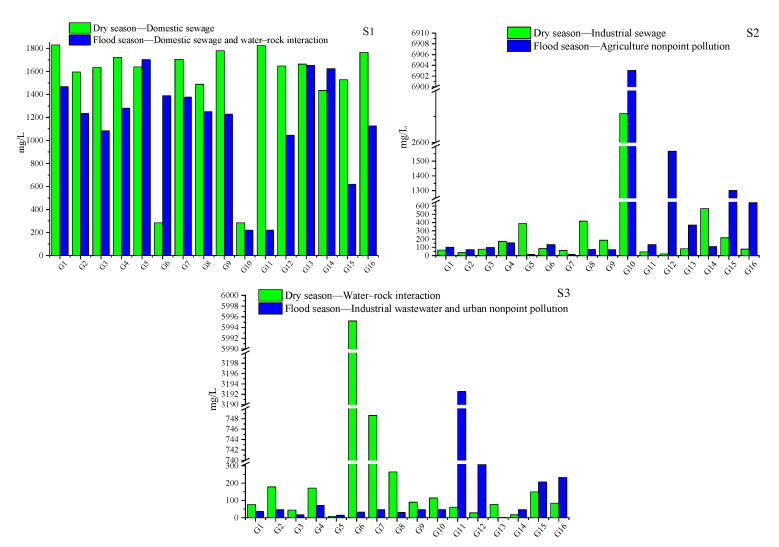
Estimated contributions (mg/L) from each source at the sampling sites during the dry and flood seasons obtained by the PMF model (Note: (**S1**): Source 1; (**S2**): Source 2; (**S3**): Source 3).

Figure 4 shows the mean contributions (mg/L) of three sources at 16 sampling sites based on the output of the PMF model. In addition, Table 5 summarized the main characteristics of each site. On the whole, the mean contribution of domestic sewage and industrial sewage to 16 sampling sites in the dry season (1489 and 322.5 mg/L) was higher than that in the flood season (1158 and 273.6 mg/L), which is mainly due to the dilution of excessive rainfall in the flood season (Figure 4S1) [18]. The contribution rate of domestic sewage during the dry and flood season was higher in the village sites (1646 and 1277 mg/L) than that in farmland sites (1589 and 1155 mg/L) and county sites (1398 and 873.2 mg/L), which may be due to the domestic sewage substandard emissions in the village region. However, the spatial variation at different land use patterns in the dry and flood season showed that the contribution rate of industrial sewage in village sites (404.2 and 384.4 mg/L) was higher than that in county sites (245.4 and 220.0 mg/L) and farmland sites (147.3 and 95.6 mg/L). In addition, in the dry season, the mean contribution of water–rock interaction at 16 sites was higher in the village sites (810.8 mg/L) than that of the county (116.6 mg/L) and farmland sites (115.5 mg/L). The mean contribution of agricultural nonpoint pollution at 16 sites in the flood season in the farmland sites (970.5 mg/L) was higher than that in the village sites (880.5 mg/L) and county sites (379.0 mg/L). It is noteworthy that the highest contribution of agricultural nonpoint pollution and urban nonpoint pollution was from site 10 (agricultural area) and site 11 (county area) (6903 and 3103 mg/L), respectively. This was closely related to the excessive application of chemical fertilizer in agricultural areas and the heavy traffic in urban areas.

#### 3.6.2. Estimated Contribution Rate (%) of Each Source to 14 Water Quality Variables

**Figure 5:**
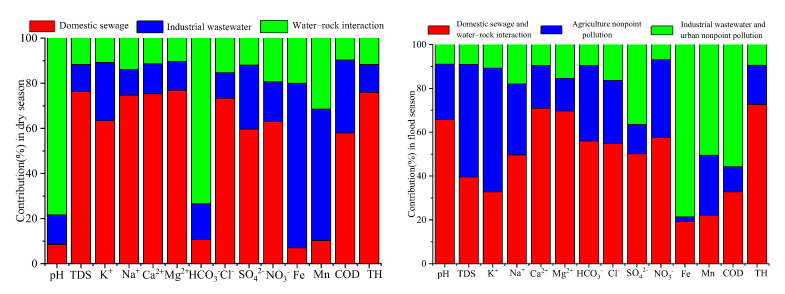
Source contribution (in %) of each variable in the dry and flood seasons in the Ye River basin.

The contribution proportion of each source to each groundwater quality parameters was calculated using the PMF model. As shown in Figure 5, in the dry season, most of the water-quality parameters were affected by domestic sewage (76.3% of TDS, 63.5% of K^+^, 74.7% of Na^+^, 75.3% of Ca^2+^, 76.9% of Mg^2+^, 73.3% of Cl^−^, 59.7% of SO_4_^2−^, 63.1% of NO_3_^−^, 58.0% of COD and 75.9% of TH) industrial sewage (73.0% of Fe, 58.2% of Mn, 32.3% of COD and 28.4% of SO_4_^2−^) and water–rock interaction (78.4% of pH and 73.4% of HCO_3_^−^ and 31.5% of Mn).

In the flood season, water-quality parameters were affected by domestic sewage and water–rock interaction (65.7% of pH, 49.7% of Na^+^, 70.9% of Ca^2+^, 69.7% of Mg^2+^, 50.1% of SO_4_^2−^, 57.6% of NO_3_^−^, 54.9% of Cl^−^, 56.0% of HCO_3_^−^, 72.7% of TH, 39.6% of TDS, and 32.8% of K^+^), agricultural nonpoint pollution (51.4% of TDS, 56.5% of K^+^, and 35.5% of NO_3_^−^), and industrial wastewater and urban nonpoint pollution (78.6% of Fe, 50.5% of Mn, 55.7% of COD, and 36.5% of SO_4_^2−^).

Based on the results of our study, the point sources (domestic sewage and industrial wastewater) remain the most critical groundwater pollution sources (especially in the dry season, where contribution proportion of point source was 77.5%) in the Ye River area of China. Therefore, local governments should act to strengthen the sewage treatment infrastructure and also pass strict legislation to prohibit the substandard discharge of sewage and wastewater. Agricultural nonpoint pollution was also an important source of groundwater contamination in the flood season; thus, local government should pursue management of fertilization strategies—such as soil formula fertilization—to increase the efficiency of nitrogen uptake by plants. Implementing the abovementioned measures in a timely way can prevent an increase in the nitrate levels in the Ye River basin.

#### 3.6.3. Uncertainty analysis

In this study, a PMF model was used to quantify the contribution of the three factors (sources) to the water-quality variables and sampling sites along the Ye River of the Hebei Province, China. However, there are some uncertainties about these results. In general, the uncertainty of solutions mainly arises from three causes: (1) random errors of the data matrix, which are introduced by measurement procedures; (2) rotational ambiguity resulting from the fact that multiple PMF solutions can have the same or very close values of object function Q; (3) modeling errors caused by the simplification of the real system [47]. To resolve this, the reliability and robustness of the results obtained from PMF model (base run) were evaluated with error estimation using the BS and DISP methods. A total of 200 run of BS resampling and PMF model fitting were performed, and the size of bootstrap data was set to 95 based on the recommendation of the PMF model. For each bootstrap run, the factors (sources) derived from PMF model were mapped to those of the base run, according to the relationship between their factor contributions. A bootstrap factor was assigned to the base factor, with which it has the lower correlation (R^2^ < 0.6), and it was considered “unmapped”. Table 6 showed that more than 85% of the base factors were reproduced, suggesting that factor profiles of the base run are reliable.

The DISP analysis could obtain the number of factors, and it is able to judge the stability of the selected PMF solution. Swaps occur when the displacements change factors significantly so that they exchange identities, suggesting that the PMF solution is not well defined [48]. In our study, there was no factor swaps observed under the lowest maximum allowable change of Q (dQ max) level. Therefore, the results of both BS and DISP suggest that the three-factor PMF solution is stable. However, the results of the contribution ratio have some uncertainty, as several basic assumptions of the PMF model are not generally applicable in many cases. For example, the influence of some ions sources of groundwater is restricted to adjacent areas, when the ions might always affect the whole area. The uncertainty ranges for the contributions of the three sources to 14 water-quality variables in the dry and flood season of the Ye River were obtained with error estimation (Table 7).

## 4. Conclusions

In this study, spatiotemporal variations in groundwater quality and pollution sources were identified along the Ye River of the Hebei Province, China, using the WQI and PMF model. Overall, the mean concentration of TH, SO_4_^2−^, and NO_3_^−^ were 626.99, 216.47, and 134.60 mg/L, respectively. Their exceeding standard rates were 78.13, 34.38, and 59.38%, respectively. The main groundwater hydrochemical type has changed from HCO_3_-Ca(Mg) to HCO_3_·SO_4_-Ca(Mg). These data indicated that the groundwater quality was generally affected by anthropogenic activities.

Spatial variation in groundwater quality was mainly affected by land use and showed that the mean concentration of NO_3_^−^ was higher in the farmland area than in the villages and county area. Temporal variation in groundwater quality was primarily controlled by rainfall, and the mean concentrations of SO_4_^2−^ and Fe were higher in the dry season than in the flood season.

Based on the results of WQI, the groundwater quality was better in the flood season than in the dry season due to the diluting effect of rainfall runoff on pollutants. Notably, the groundwater quality of the farmland area was relatively poor because it was affected by multiple pollution sources.

The PMF model results showed that the major groundwater pollution sources were domestic sewage (52.4%), industrial wastewater (24.1%), and enhanced water–rock interaction induced by intensely exploited groundwater (23.6%) in the dry season. Meanwhile in the flood season, they were domestic sewage and water–rock interactions (49.6%), agricultural nonpoint pollution (26.1%), and industrial wastewater and urban nonpoint pollution (24.0%). The mean contribution of the domestic sewage and industrial sewage to 16 sampling sites in the dry season (1489 and 322.5 mg/L, respectively) was higher than that in the flood season (1158 and 273.6 mg/L, respectively). To sum up, the point sources (domestic sewage and industrial wastewater) remain the most critical groundwater pollution sources in this region. These results indicated that the local governments urgently need to develop a priority strategy to control nitrate contamination and achieve water resource sustainability in the Ye River area. In addition, this study was conducted within one hydrological year; thus, the results of the study may have some uncertainty. Therefore, future studies should carry out a long-time series sampling strategy to further confirm the accuracy of the results.

## Figures and Tables

**Table 1:** Relative weight of physicochemical parameters and water quality standard (all units of the parameters are mg/L except pH).

Parameters	Water Quality Standards	Weight (*W_i_*)	Relative Weight (*RW_i_*)
pH	6.5–8.5	4	0.082
TDS	1000	5	0.102
Na^+^	200	3	0.061
Ca^2+^	75	3	0.061
Mg^2+^	50	3	0.061
Cl^−^	250	5	0.102
SO_4_^2−^	250	5	0.102
HCO_3_^−^	500	1	0.020
NO_3_^−^	88.6	5	0.102
Fe	0.3	3	0.061
Mn	0.1	3	0.061
COD	3.0	5	0.102
TH	450	4	0.082
Sum		58	1

Note: The Mg^2+^, Ca^2+^ and HCO_3_^−^ refer to the World Health Organization (2011) standards, the other parameters refer to the grade III standard for groundwater quality in China (GB/T 14848-2017).

**Table 2:** Descriptive statistics of groundwater quality parameters along the Ye River.

Parameters (N = 32)	Units	Range	Average	S.D.	Standard	Below Standardsfor All Sites (%)
pH	-	6.91–7.87	7.37	0.22	6.5–8.5	0
DO	mg/L	2.67–9.45	6.62	1.67	-	-
TDS	mg/L	499.23–1461.40	866.80	259.10	1000	31.25
K^+^	mg/L	0.55–5.67	2.22	1.29	-	-
Na^+^	mg/L	8.88–174.97	42.17	28.92	200	0
Ca^2+^	mg/L	106.62–324.65	186.08	59.83	-	-
Mg^2+^	mg/L	11.42–88.35	39.27	20.08	-	-
HCO_3_^−^	mg/L	176.20–462.10	312.32	78.94	-	-
Cl^−^	mg/L	25.53–280.80	96.38	62.32	250	9.38
SO_4_^2−^	mg/L	69.20–342.30	216.47	66.79	250	34.38
NO_3_^−^	mg/L	15.07–376.50	134.60	100.32	88.6	59.38
Fe	mg/L	0.011–0.998	0.129	0.199	0.3	6.25
Mn	mg/L	0.001–0.045	0.006	0.011	0.1	0
COD	mg/L	0.36–1.41	0.78	0.31	3.0	0
TH	mg/L	370.79–1091.00	626.99	194.33	450	78.13

Note: N is the number of samples; standard is grade III standard for groundwater quality in China (GB/T 14848-2017).

**Table 3:** Water quality classification of different seasons along the Ye River.

WQI Range	Dry Season		Flood Season	
	Number of Samples	Percentage of Samples (%)	Number of Samples	Percentage of Samples (%)
Excellent water	1	6.2	1	6.2
Good water	9	56.3	14	87.5
Poor water	6	37.5	1	6.3
Very poor water	0	0	0	0
Water unsuitable for drinking purposes	0	0	0	0
Sum	16		16	

**Table 4:** Source profiles obtained from the PMF model.

Parameters	Dry Season			Flood Season		
	Factor 1	Factor 2	Factor 3	Factor 1	Factor 2	Factor 3
pH	0.63	0.96	5.75	4.74	1.83	0.65
TDS	597.69	94.13	91.27	294.79	382.46	67.19
K^+^	0.85	0.35	0.14	0.41	0.71	0.14
Na^+^	24.19	3.69	4.49	10.83	7.07	3.91
Ca^2+^	122.94	21.69	18.57	108.66	29.99	14.56
Mg^2+^	21.71	3.60	2.93	16.12	3.46	3.56
HCO_3_^−^	31.75	47.35	218.73	151.88	93.43	25.93
Cl^−^	39.12	6.09	8.13	35.09	18.36	10.46
SO_4_^2−^	123.51	58.81	24.68	72.13	19.29	52.45
NO_3_^−^	28.01	7.82	8.58	18.75	11.54	2.24
Fe	0.01	0.07	0.02	0.02	0.00	0.10
Mn	0.001	0.003	0.002	0.001	0.002	0.003
COD	0.39	0.22	0.06	0.27	0.09	0.45
TH	426.75	69.75	65.66	389.49	95.70	50.81
Possible sources	Domestic sewage	Industrial sewage	Water–rock interaction	Domestic sewage and water–rock interaction	Agriculture nonpoint pollution	Industrial wastewater and urban nonpoint pollution
Contribution (%)	52.37	24.12	23.51	49.55	26.12	23.94

**Table 5:** Statistic table of the main characteristics of each site.

Sites	Land Use	Depth of the Well (m)	Depth of Groundwater (m)	Pollution Sources
G01	Village	20	9.6	Sewage and Manure
G02	Agriculture	30	10.5	Fertilizer
G03	Village	40	12.5	Sewage and Manure
G04	County	35	16.6	Sewage and coal mine effluent
G05	Village	18	10.3	Sewage and wastewater
G06	Village	33	15.1	Sewage
G07	Village	15	3.2	Sewage and coal mine effluent
G08	Agriculture	25	18.5	Fertilizer and sewage
G09	Agriculture	20	12.2	Fertilizer and Manure
G10	Village	12	6.5	Sewage and wastewater
G11	Village	28	15.3	Sewage and manure
G12	Agriculture	22	14.8	Fertilizer
G13	Village	12	8.7	Sewage
G14	Agriculture	25	9.5	Fertilizer
G15	County	50	22.5	Sewage
G16	County	45	23.4	Sewage

**Table 6:** Mapping of bootstrap factors to base factors derived from PMF model.

Bootstrap	Dry Season				Flood Season			
	Factor 1	Factor 2	Factor 3	Unmapped	Factor 1	Factor 2	Factor 3	Unmapped
Factor 1	195	4	1	0	190	7	3	0
Factor 2	8	188	4	0	9	186	5	0
Factor 3	3	5	192	0	3	4	193	0

**Table 7:** Results of uncertainty analysis for factor contributions ratio (%) to 14 water-quality parameters in the Ye River of Hebei Province, China using the error estimation methods of displacement of factor elements (DISP).

Parameters	Factor 1				Factor 2				Factor 3			
	Dry Season		Flood Season		Dry Season		Flood Season		Dry Season		Flood Season	
	Mean	SD	Mean	SD	Mean	SD	Mean	SD	Mean	SD	Mean	SD
pH	8.5	5.6	65.7	7.3	13.1	5.2	25.3	4.7	78.4	3.2	9.0	3.7
TDS	76.3	4.2	39.6	7.6	12.0	2.9	51.4	5.0	11.7	4.1	9.0	3.5
K^+^	63.5	3.6	32.8	5.5	25.8	4.8	56.4	4.2	10.7	3.0	10.8	2.2
Na^+^	74.7	4.6	49.7	7.1	11.4	4.7	32.4	4.6	13.9	3.1	17.9	3.6
Ca^2+^	75.3	5.0	70.9	6.4	13.3	5.0	19.6	4.6	11.4	3.1	9.5	2.6
Mg^2+^	76.9	4.7	69.7	6.7	12.8	4.3	15.0	4.4	10.4	3.3	15.4	3.5
HCO_3_^−^	10.7	3.4	56.0	7.0	15.9	4.7	34.4	4.5	73.4	3.0	9.6	3.7
Cl^−^	73.3	3.5	54.9	6.6	11.4	5.4	28.7	4.7	15.2	2.9	16.4	2.6
SO_4_^2−^	59.7	3.1	50.1	4.8	28.4	5.5	13.4	3.7	11.9	3.8	36.5	2.8
NO_3_^−^	63.1	4.5	57.6	7.2	17.6	4.9	35.5	4.7	19.3	3.0	6.9	3.7
Fe	7.0	7.3	19.2	2.1	73.0	14.0	2.3	6.8	20.0	9.6	78.6	6.0
Mn	10.3	13.4	22.1	11.7	58.2	14.6	27.4	7.3	31.5	6.7	50.5	8.4
COD	58.0	4.5	32.8	7.3	32.3	2.9	11.5	4.8	9.7	4.6	55.7	3.5
TH	75.9	4.6	72.7	7.2	12.4	4.9	17.9	4.7	11.7	3.1	9.5	3.6

Note: SD: standard deviation.

## Data Availability

Not applicable.
